# Medical students' dilemma during the Covid-19 pandemic; between the will to help and the fear of contamination

**DOI:** 10.1080/10872981.2020.1784374

**Published:** 2020-06-24

**Authors:** Ghita Hjiej, Maryam Fourtassi

**Affiliations:** a Faculty of Medicine of Oujda, Mohammed Premier University, Oujda, Morocco; b Laboratory of Epidemiology, Clinical Research and Public Health, Mohammed Premier University, Oujda, Morocco

**Keywords:** COVID-19, Morocco, Medical students

Because of the Coronavirus disease (COVID-19) outbreak, the world has been facing an unprecedented health and economic crisis [1]. In Morocco, many extreme measures have been taken by authorities to better contain the spread of the virus, including enforced social distancing and imposed home confinement to everyone but some necessary exceptions [2]. And whilst most people are locked down in their homes, limited to telecommuting and distance working, health workers, doctors and nurses are mobilized in hospitals dealing with infected and sick patients.

However, and despite the need for every possible help to the healthcare teams, medical students were denied access to hospitals and their clinical rounds have been suspended in order to protect them and their families. This situation has generated an emotional conflict amongst medical students, not knowing exactly what they are supposed to do, and what should be expected of them towards their communities as part of the health workers’ teams.

Some of the very recently common conversations in medical students’ groups in social media raise this recurrent emotional dilemma between the will to return to hospitals and fulfil what they consider as their primary duty; caring for patients, and the necessity to respect the lockdown measures and stay at home to avoid being transmission vectors of the coronavirus. Being forced to stay away and not participate in the battlefield was generally felt as painfully frustrating. But how can medical students be helpful to their societies without being directly engaged in clinical practice, during this COVID-19 pandemic?

Hospitals are not the only location where a person with some basics of medical knowledge can be useful and effectively take part in managing this current health crisis, and prevent an even worse disaster from happening. For example, medical students can play a major role in raising awareness within less informed individuals in order to improve their acceptance and implementation of the already recommended preventive measures. Medical students are more reliable in conducting a fact search regarding the pandemic. They can correct the widely spread misleading information and share the verified and pertinent information with other people in their close environment or on a larger scale through social media. Moreover, medical students can also volunteer to help doctors in the medical regulation centres to answer calls from worried people and provide guidance and orientation based on their symptoms and following pre-established guidelines. Medical students should keep in mind that even if they are at an advanced stage of training, and might have the necessary knowledge needed for clinical practice, they still have to graduate to take the responsibility of the oath. Even if they are dying to help onsite, it’s neither their place nor their current responsibility. So, the best way to help their fellow citizens would be by participating in the other aspects of the fight against the virus, and be patient, because they might be joining the battlefield sooner than expected.
